# The Revolution in Viral Genomics as Exemplified by the Bioinformatic Analysis of Human Adenoviruses

**DOI:** 10.3390/v2071367

**Published:** 2010-06-28

**Authors:** Sarah Torres, James Chodosh, Donald Seto, Morris S. Jones

**Affiliations:** 1 Clinical Investigation Facility, David Grant USAF Medical Center, Travis AFB, CA 94535, USA; E-Mail: sarah.torres1@travis.af.mil; 2 Department of Ophthalmology, Howe Laboratory, Massachusetts Eye and Ear Infirmary, Harvard Medical School, Boston, 02114 MA, USA; E-Mail: James_Chodosh@meei.harvard.edu; 3 Department of Bioinformatics and Computational Biology, George Mason University, Manassas, VA 20110, USA; E-Mail: dseto@gmu.edu

**Keywords:** human adenoviruses, genomics, bioinformatics

## Abstract

Over the past 30 years, genomic and bioinformatic analysis of human adenoviruses has been achieved using a variety of DNA sequencing methods; initially with the use of restriction enzymes and more currently with the use of the GS FLX pyrosequencing technology. Following the conception of DNA sequencing in the 1970s, analysis of adenoviruses has evolved from 100 base pair mRNA fragments to entire genomes. Comparative genomics of adenoviruses made its debut in 1984 when nucleotides and amino acids of coding sequences within the hexon genes of two human adenoviruses (HAdV), HAdV–C2 and HAdV–C5, were compared and analyzed. It was determined that there were three different zones (1–393, 394–1410, 1411–2910) within the hexon gene, of which HAdV–C2 and HAdV–C5 shared zones 1 and 3 with 95% and 89.5% nucleotide identity, respectively. In 1992, HAdV-C5 became the first adenovirus genome to be fully sequenced using the Sanger method. Over the next seven years, whole genome analysis and characterization was completed using bioinformatic tools such as blastn, tblastx, ClustalV and FASTA, in order to determine key proteins in species HAdV-A through HAdV-F. The bioinformatic revolution was initiated with the introduction of a novel species, HAdV-G, that was typed and named by the use of whole genome sequencing and phylogenetics as opposed to traditional serology. HAdV bioinformatics will continue to advance as the latest sequencing technology enables scientists to add to and expand the resource databases. As a result of these advancements, how novel HAdVs are typed has changed. Bioinformatic analysis has become the revolutionary tool that has significantly accelerated the in-depth study of HAdV microevolution through comparative genomics.

## 1. Introduction

Genomics and bioinformatics have provided a highly detailed understanding of organisms through the analysis of primary sequence data. With the rapid adoption and continuing improvement of next generation DNA sequencing technology and methodology, understanding the biology and pathology of human pathogens is tantalizingly within reach. Viruses, especially human adenoviruses, are an important model for defining both the biology and pathology based on the primary sequence data, due to their genome size and importance as human pathogens. In this review we provide a historical perspective of how the genomic and bioinformatic analyses of human adenoviruses (HAdVs) have advanced in the last 30 years. We start by describing the first few nucleotides that were sequenced using both the Maxam-Gilbert and the Sanger sequencing methods, then the first complete genes as well as the first whole adenovirus genome to be assembled and analyzed. Initially, the genomes were sequenced in parts from different laboratories and using different methods, and assembled as a complete but mosaic contribution. As noted, some of these data, including whole genomes, for example HAdV-D17, contain sequencing errors that hinder comparative genomic analysis. Currently, technologies such as the GS FLX pyrosequencing method provide definitive single laboratory-initiated and single method high-quality genomes. The major advances in the bioinformatic analysis of adenoviruses will also be discussed, with highly detailed snapshots of human adenoviruses in general, pathology and molecular evolution of new types and emerging or re-emerging pathogens. This review ends with a description of the current state of adenovirus bioinformatics and developing trends.

## 2. The Labor of Human Adenovirus Genomics

In the beginning of adenoviral genomics, positions within the human adenovirus (HAdV) type 2 genome were mapped using restriction enzyme analysis [1]. A few years later, upon the advent of DNA sequencing by Maxam and Gilbert [2], 139 base pairs of the mRNA leading up to the 5′ region of the hexon gene was determined [3]. Benefits of this work included visualization of the two-dimensional nucleotide structure of the HAdV-C2 promoter, and a hypothetical determination of the secondary structure in the associated mRNA.

### 

#### First Example of Comparative Genomics in Human Adenoviruses

**Figure 1:**
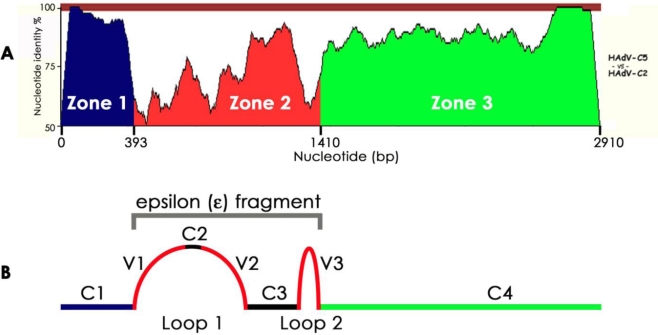
Analysis of the hexon gene. **(a)** A Nucleotide homology between the hexon coding sequences of HAdV-C2 and HAdV-C5. zPicture was used to compare the whole genomes for regions of homology/similarity. Along the *y*-axis, the baseline is set at 50% nucleotide identity and the top line is set at 100%; the entire gene length is displayed on the *x*-axis. **(b)** Schematic of the hexon protein. Variable regions V1, V2, and V3 are located in loops 1 and 2 and are outwardly oriented on the surface of the adenovirus particle. The epsilon (ε) fragment includes regions V1, C2, V2, C3, and V3.

As an early demonstration of comparative genomics, the hexon genes of HAdV-C2 and HAdV-C5 were the first two human adenovirus genes to be compared to one another [4]. In that study, analysis of the nucleotides and amino acids of the two hexon coding sequences demonstrated that the hexon gene could be divided up into three different zones (1–393, 394–1410, 1411–2910). Zones 1 and 3 in the hexon genes of HAdV-C2 and HAdV-C5 had 95% and 89.5% nucleotide identity, respectively (Figure 1A) [4]. The nucleotide identity of the two genes diverged in zone 2 (Figure 1A). Today, zone 2 is recognized as the ε determinant, which contains the type-specific epitopes of the hexon gene (Figure 1B).

## 3. The Birth of Human Adenovirus Genomics

### 3.1. HAdV-C2: The First HAdV Sequenced

**Figure 2:**
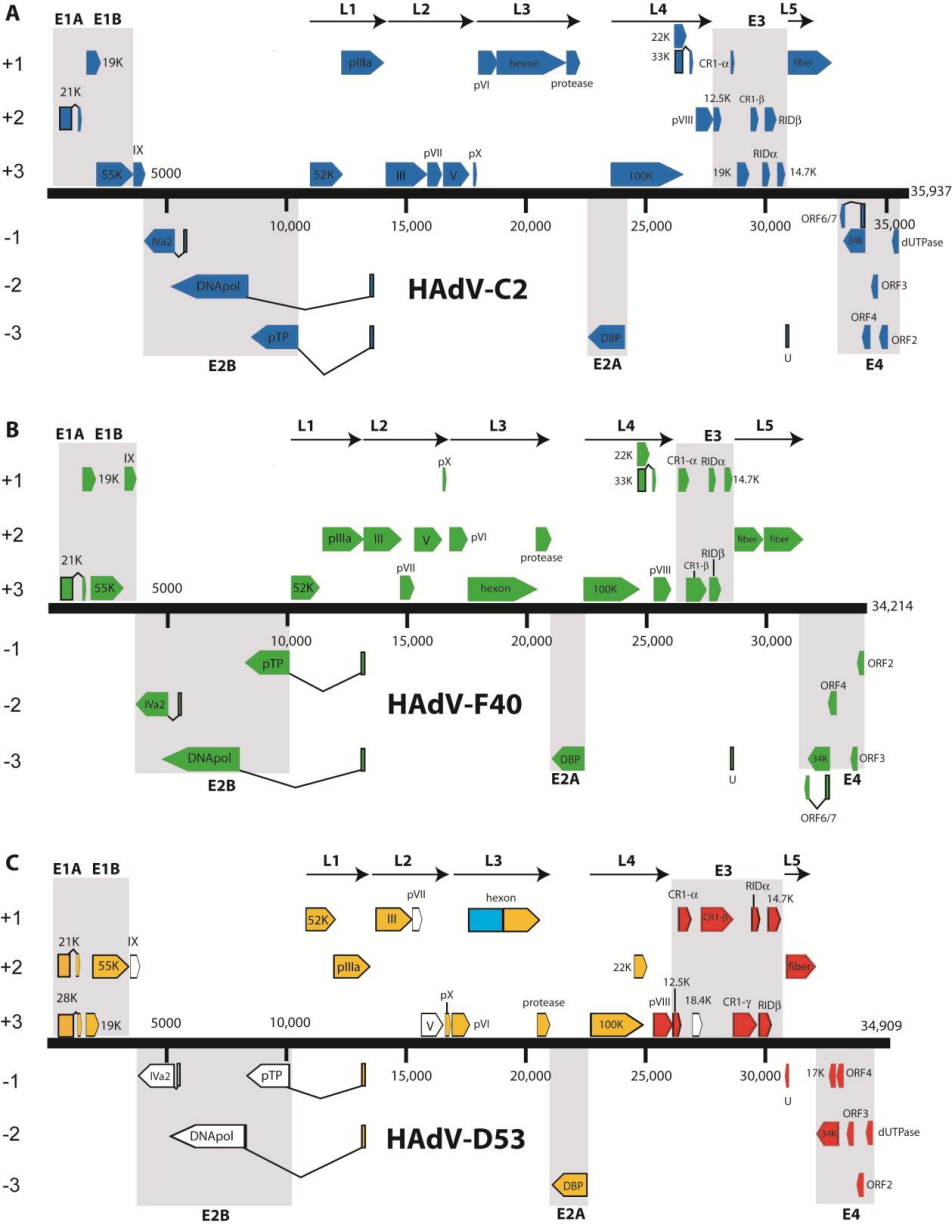
Transcriptional map and genome organization of HAdV-C2, HAdV-F40, and HAdV-D53. Genomes are represented by a central black horizontal line marked at 5-kbp intervals. Protein encoding regions are shown as boxes. Boxes above the black line represent open reading frames (ORFs) that are encoded on the forward (or upper) strand. Boxes underneath the black line represent ORFs that are encoded on the reverse (or lower) strand. **(a)** HAdV-C2. **(b)** HAdV-F40. **(c)** HAdV-D53. Figure 2B was adapted from Walsh *et al*., 2009 with permission [6].

Human adenovirus genomics was born in 1984 when Roberts and coworkers amalgamated pieces of the HAdV-C2 genome, sequenced in separate laboratories during different time periods, and with dissimilar methods, to present whole and complete genome analysis (Figure 2A) [5]. Prior to the entire HAdV-C2 sequence being deduced as 35,937 nucleotides, the locations of genes in adenovirus genomes were described using a map unit system [1]. After the HAdV-C2 genome was analyzed, a coordinate system was introduced where 1% of the genome equaled 365 nucleotides [5]. However, since adenovirus genomes differ in length, this methodology did not stand the test of time.

### 3.2. HAdV-C5

Although it was a piecemeal effort, eight years later, the whole genome of HAdV-C5 was determined using Sanger sequencing (Table 1). This accomplishment was significant because it allowed for direct genomic comparison between two adenoviral genomes [7]. Comparative analysis of the HAdV-C2 and HAdV-C5 genomes demonstrated that the genes with the greatest amino acid differences were the hexon, fiber, and the 19K ORF located in the E3 gene. This has since been confirmed and updated by pairwise alignment of multiple HAdV genomes from all known species sequenced to date [8].

In the next seven years, three adenovirus genomes (HAdV-F40, HAdV-A12, HAdV-D17) were sequenced and deposited in GenBank (Table 1). Bioinformatic analysis of the HAdV-F40 genome revealed several features not seen previously in a human adenovirus: two fiber genes and the U protein (Figure 2B) [9]. The percent identities of key proteins in species HAdV-A through HAdV-F, as well as other animal adenoviruses, were compared. Since HAdV-A12 was the first whole genome to be sequenced from species HAdV-A (Table 1), Sprengel and colleagues compared it to HAdV-C2. Comparison of the HAdV-A12 and HAdV-C2 genomes was innovative in that it was the first instance where powerful bioinformatic tools such as blastn, tblastx, ClustalV, and FASTA were used on whole adenovirus genomes [10]. The use of ClustalV was ground-breaking in that it allowed researchers the opportunity to view the overall similarity of two genomes, on the nucleotide level, in one figure [10]. This led to the finding that the HAdV-A12 and HAdV-C5 genomes have the highest nucleotide identity in the 5000–8000 base pair (bp) region (DNA polymerase). The remaining members of species HAdV-A, HAdV-A18 and HAdV-A31, were recently sequenced and analyzed [11,12].

### 3.3. Illegitimate Recombination Offered as a Possible Mechanism for Molecular Evolution

In 1996, Crawford-Miksa and Schnurr postulated that adenovirus molecular evolution is expedited by illegitimate recombination in the hexon gene of human adenoviruses due to slippage of the HAdV DNA polymerase in repetitive polypurine stretches [13]. This provided a plausible model as to how a species of viruses with a stable DNA genome has the potential to evolve over a short period of time. Bioinformatic analysis has revealed that the neutralization determinants are located in two loops that cover the surface of adenoviruses in the hexon gene (Figure 1B) [13,14] and that recombination is highly structured [6,15–17]. These studies were important because they helped scientists visualize how human adenoviruses could evolve.

### 3.4. HAdV-B11

In 2003, the HAdV-B11 genome was the first HAdV from species HAdV-B to be completely sequenced [18,19]. As human adenoviruses are important vectors in human gene therapy trials, other HAdV genomes have been sequenced and archived in GenBank. The HAdV-B11 genome studies marked the first time that phylogenetic analysis was used to examine the relationships between homologous genes from all HAdV species.

Traditionally, HAdVs, as highly contagious respiratory pathogens, have been of immediate concerns to military populations, particularly basic training personnel [20,21]. In the 1970s, vaccines against HAdV types 4 and 7 were produced, validated and deployed [22,23]. These vaccines were successful in preventing respiratory diseases, particularly acute respiratory disease outbreaks in the U.S. for over 20 years [22,23]. The manufacture and deployment of these vaccines were discontinued in the 1990s. Soon after production of vaccines against HAdV-E4 and HAdV-B7 ceased in 1997, an outbreak of HAdV-E4 erupted at Fort Jackson [24], causing unexpected and highly contagious acute respiratory disease. The re-emergence of epidemic outbreaks of febrile respiratory infections caused by infection with HAdV-E4 and HAdV-B7 among basic military trainees was of immediate pressing concern to the U.S. Department of Defense, which led to the creation of the Epidemic Outbreak Surveillance (EOS) Consortium, as a program funded in part and sponsored by the Office of the U.S. Air Force Surgeon General. As part of an effort to redevelop the HAdV-E4 and HAdV-B7 vaccines and to develop molecular surveillance platforms for respiratory disease pathogen detection, the EOS Consortium sequenced 17 genomes of HAdVs, including the vaccine strains for HAdV-E4vac and HAdV-B7vac [25] as well as both the HAdV-E4 [26] and HAdV-B7 reference genome [25]. To gain a greater understanding of HAdVs in the species HAdV-B, EOS also sequenced prototypes HAdV-C1 [27], HAdV-B3, HAdV-B16, HAdV-B21, HAdV-B34, HAdV-B50 and HAdV-C6, more than doubling the number of completely sequenced genomes at the time. Additional contemporary circulating field strains of aforementioned HAdVs were also sequenced.

Analysis of the HAdV-E4vac and HAdV-B7vac genomes was a turning point in bioinformatic and genomic investigation, because the genomes of both viruses were compared and contrasted with field strains within the same species as opposed to HAdV-C2 or HAdV-C5, with a fine nucleotide-level granularity. Additionally, the implications for all insertions/deletions (indels), larger gaps, and single base substitutions in HAdV-E4 and HAdV-B7 were discussed [26].

In 2006, five additional HAdV genomes (HAdV-F41, HAdV-D26, HAdV-D46, HAdV-D48, HAdV-D49) were sequenced and posted in GenBank (Table 1). However, their genomes were not fully analyzed in peer-reviewed articles. Nevertheless, their availability in GenBank allows their use for comparison to other HAdV genomes.

## 4. Major Advances in the Bioinformatic Revolution of Human Adenoviruses

### 4.1. HAdV-G52

The discovery of HAdV-G52 marked the beginning of the bioinformatic revolution in understanding human adenoviruses from a primary sequence perspective. Not only was this event significant because a new species (HAdV-G) was introduced, but it was the first time a new adenovirus type and species were proclaimed by whole genome sequencing, as opposed to serology [32]. However, this was not without controversy as the announcement of the first adenovirus discovery in the 21st century was challenged, albeit unsuccessfully [38]. Not only has HAdV-G52 been accepted as the 52nd type of human adenovirus, but the new species HAdV-G has also been accepted by the International Congress of Virology and Taxonomy.

### 4.2. Simplot Analysis of Novel Adenovirus in Species HAdV-C

Lukashev and coworkers were the first group to use bootscan and Simplot analysis to analyze partially sequenced adenovirus genomes for recombination. Using these powerful tools, they found that recombination is frequent in adenovirus species HAdV-B, -C, and -D [15]. Interestingly, they found one clinical isolate (strain #16700) from species HAdV-C that had high amino acid identity to the fiber gene of HAdV-C6 and a hexon gene that was distinct from all viruses in species HAdV-C [15]. Moreover, strain #16700 could not be serotyped unambiguously in a neutralization test and most likely represents a novel type within species HAdV-C. Bootscan analysis of the 3′ end of the #16700 strain genome (nucleotides 18838–33452) demonstrated multiple recombination events. However, the location of the recombination breakpoints was not pinpointed.

### 4.3. HAdV-D53

**Figure 3:**
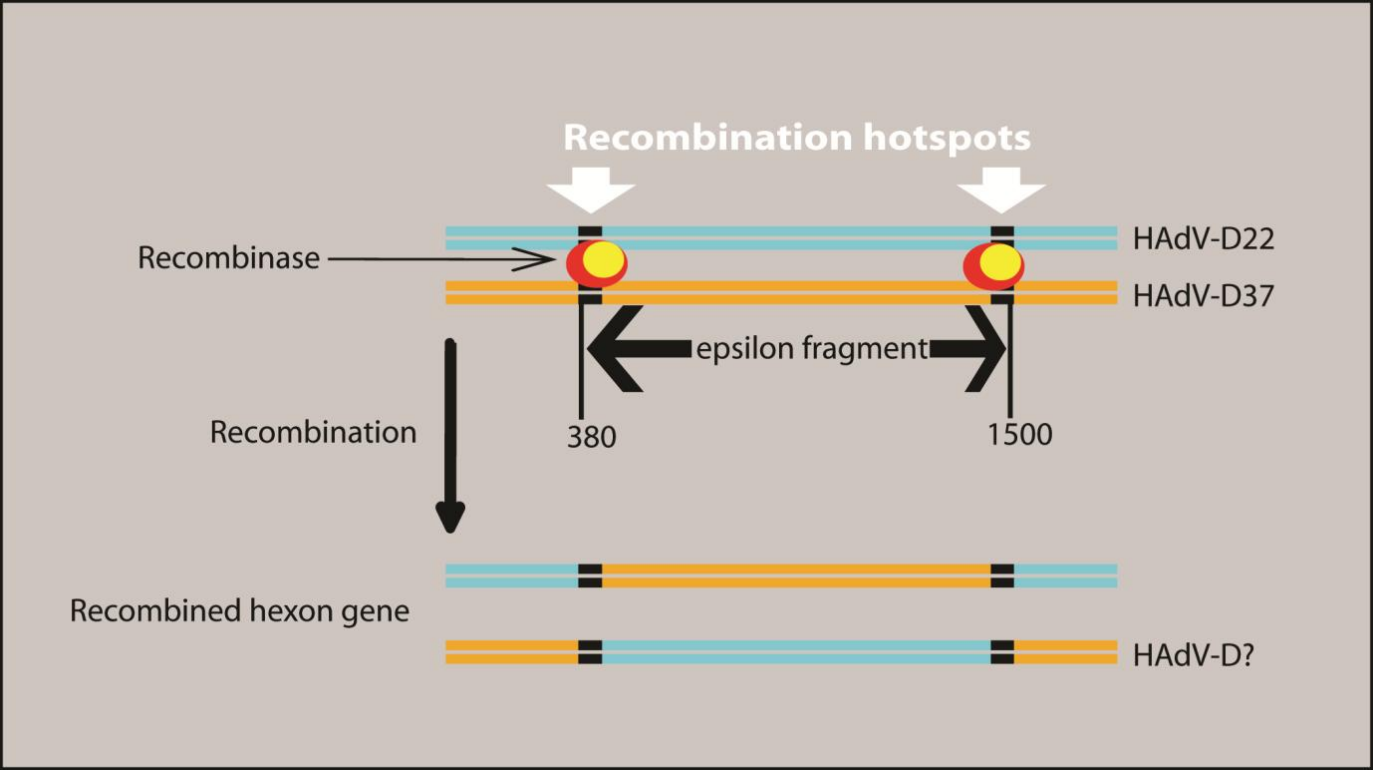
Model of recombination for human adenoviruses. Recombination in the hexon gene of HAdVs is hypothesized to occur around nucleotide positions 380 and 1500. These positions frame the neutralization determinants (epsilon ε fragment) of HAdVs. These positions are also demarcated by white arrows. The indicated nucleotide positions are based on HAdV-D53 [6].

In the previous decade it was cost-prohibitive to sequence entire adenoviral genomes; thus, to properly declare a novel adenovirus, one had to use all of the known antisera that reacted against the known adenoviruses [39]. When an adenovirus was neutralized by antisera dissimilar to its hemagglutination profile, it was erroneously named an intermediate recombinant [40]. Without the complete genome sequence it was impossible to know where putative recombination events occurred. Today, sequencing a complete adenovirus genome is much cheaper and more efficient, and is of higher quality than in 1984. Bioinformatic analysis of fully sequenced recombinant adenoviruses has made it possible to determine the exact loci of recombination. Such was the case when HAdV-D53 was characterized using genomics and bioinformatics [6]. HAdV-D53 was initially described as intermediate HAdV-D22.H8, because partial sequencing of the hexon gene revealed 100% identity to the HAdV-D22 hexon sequence and it was assumed that the rest of the genome would yield HAdV-D22 as well. The H8 was ascribed to this proposed intermediate when the fiber sequence was found to be identical to that of HAdV-D8 [40]. To elucidate the origin of the novel sequence, Walsh and colleagues sequenced the whole genome and found adenoviral sequences from known, as well as unknown (or as yet unsequenced), adenoviruses (Figure 2C) [6]. In the same study it was also shown that recombination within the hexon gene is common in species HAdV-D and that HAdV-D53, as an emergent, highly contagious keratoconjunctivitis-causing pathogen, was created by multiple recombination events. Specifically, it was shown that the recombination events in the hexon occur at the beginning and the end of the epsilon fragment (Figure 3).

### 4.4. Recombination is Common in the Penton Gene of Species HAdV-D

Recently the genome of HAdV-D22 was completely sequenced. Analysis of the penton gene confirmed that there were recombination events in two external hypervariable loops in the penton coding sequence, encompassing nucleotides 400–600 and 750–1350, which are located on the exposed area of the viral capsid [16]. These recombination events were also seen in the penton genes of HAdV-D9 and HAdV-D26, demonstrating that the aforementioned areas represent recombination hot spots within species HAdV-D.

### 4.5. Hypothetical Recognition Sites for Recombination in the Hexon Gene

**Figure 4:**
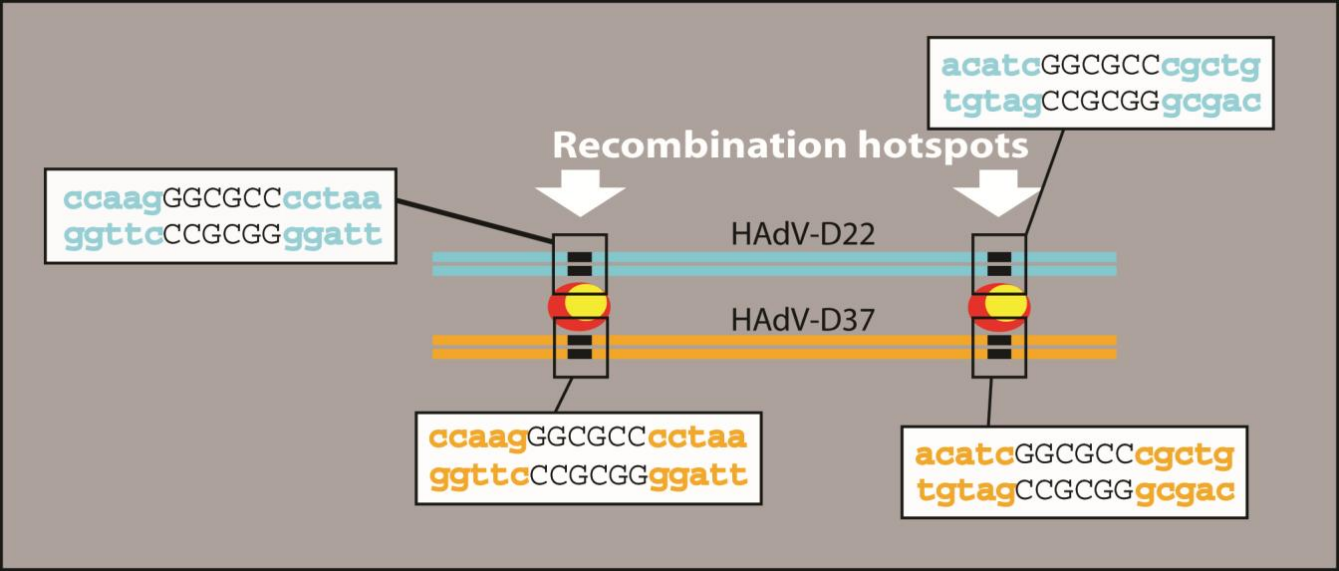
Hypothetical recognition sites for recombination in the hexon gene. Rectangular boxes represent hypothesized recognition sites, which are recognized by hypothetical recombinase. Red and yellow ovals are hypothesized recombinase proteins.

It is possible that human recombinase proteins have a propensity to bind certain sequences in adenovirus genomes. After analyzing aligned HAdV-D hexon sequences, we saw a motif that was common at the beginning and end of the epsilon fragment (Figure 4). Because we do not have biological data to confirm this observation, we acknowledge that further studies will be required to prove such a hypothesis.

### 4.6. HAdV-B55

**Figure 5:**
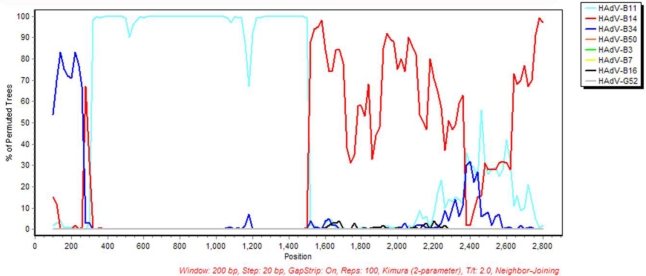
Fine-resolution bootscan analysis demonstrating recombination in the partial hexon gene of HAdV-B55. Bootscan analysis of the hexon gene shows that the proximal region, containing Loops 1 and 2, is derived from HAdV-B11. The distal portion is derived from HAdV-B14, as is the remainder of the HAdV-B55 genome. Parameters are as follows: 200-bp window; 20-bp step; 100 repetitions; neighbor-joining algorithm. This figure was adapted from Walsh *et al.,* 2010 with permission [17].

Currently, there are 55 recognized types of HAdVs [17,41]; the term “type” is used to include original serotypes 1–51, determined using traditional serology, as well as the additional four genotypes, characterized using genomics and bioinformatics. HAdV-B55, provisionally named HAdV-B11.QS, was isolated in China from a patient who died of an acute respiratory disease-induced multiple organ failure [42]. HAdV-B55 was recognized as a new human adenovirus when Walsh *et al*. presented comprehensive nucleotide sequence evidence that the HAdV-B11.QS genome was actually 97.4% identical to HAdV-B14, with the remaining 2.6% derived from HAdV-B11. Further analysis demonstrated that the only part of the HAdV-B11.QS genome that had high homology to HAdV-B11 was the epsilon fragment in the hexon gene (Figure 5), accounting for the initial serological-based characterization as HAdV-B11.

## 5. The Future

Further advancements in HAdV bioinformatics are expected as sequencing technology provides us a large number of genomes as resources. We expect these advances to develop our understanding of the microevolution of HAdVs. For example, the GS-FLX is currently able to sequence 400–600 million bases per run. With the recent publication of HAdV-D36 [37], there are only 20 remaining HAdV types (720 kb of viral genomic DNA) still unsequenced, all within species HAdV-D. Theoretically, all of the unsequenced HAdVs could be completely sequenced in one run on the GS FLX Titanium with 833-fold coverage of each genome, ensuring high quality data.

Advances in sequencing technology have also led to necessary changes in HAdV nomenclature. All of the HAdVs that have been discovered/characterized in the 21st century have been numbered without utilizing exhaustive serology [6,17,32,41]. Novel HAdVs should now be named in one of two ways. If a new HAdV is suspected, the whole genome should be sequenced and analyzed via phylogenetic analysis. If the proposed HAdV consistently falls into a different lineage than all other HAdV species, as was the case for HAdV-G52, then identification of a new HAdV species and number is justified. If phylogenetics place the virus in an existing species, but it is sufficiently distinct from other members of that species, and/or recombinant in a way that impacts the tropism or extent of disease, the virus would be considered novel and deserving of a new type number. A second method is traditional serology: if the proposed new virus cannot be neutralized by any of the known antisera, then it can be considered as novel and assigned a new number. Ideally, the characterization of any HAdV as new should include whole genome sequencing and bioinformatics analysis.

## 6. Conclusions

Advances in sequencing technology have increased the number of human adenoviruses that are currently available for comparative analysis. These advances have elucidated our understanding of the molecular evolution of HAdVs. Our clearer comprehension of HAdV genomics has also given birth to changes in how we type and number novel HAdVs. Since 35 of 55 HAdV genomes have been fully sequenced and analyzed, we know that (a) much of the HAdV genome is conserved, (b) the non-conserved regions are restricted to the penton, hexon, E3, and the fiber genes, and that (c) recombination is a common occurrence at critical loci in the penton and hexon genes of viruses in species HAdV-D. We do not know which enzymes are involved in facilitating recombination, the specific sequences that are recognized, why recombination appears to occur at a faster rate in species HAdV-D than other species, or the loci of other recombination hot spots within the HAdV genome. The characterization and discovery of novel HAdVs, as well as the genetic analysis of the 20 HAdVs which remain to be sequenced (Table 2), will increase our understanding of HAdV genomics.

## Figures and Tables

**Table 1:** Timeline of HAdV sequencing.

**HAdV**	**Year sequenced**	**Reference**

HAdV-C2	1984	[5]

HAdV-C5	1992	[7]

HAdV-F40	1993	[9]

HAdV-A12	1994	[10]

HAdV-D17	1999	NP

HAdV-B11	2003	[18]
		[19]
HAdV-B35		[28]
		[29]

HAdV-C1	2004	[27]
HAdV-D9		NP
HAdV-B16		NP
HAdV-B21		NP
HAdV-B34		NP
HAdV-B50		NP

HAdV-E4	2005	[26]
HAdV-B7		[26]
HAdV-F41		NP

HAdV-B3	2006	[30]
		[31]
HAdV-D26		NP
HAdV-D46		NP
HAdV-D48		NP
HAdV-D49		NP

HAdV-G52	2007	[32]

HAdV-D37	2008	[8]
HAdV-D19		[33]

HAdV-B14	2009	[34]
HAdV-D8		NP
HAdV-D22		[16]
HAdV-D28		NP
HAdV-A31		[35]
HAdV-D53		[6]
HAdV-D54		[36]

HAdV-A18	2010	[12]
HAdV-D36		[37]
HAdV-B55		[17]

NP – not published.

**Table 2:** Human adenoviruses that have not been completely sequenced.

**HAdV**	**ATCC number**
HAdV-D10	VR-1087
HAdV-D13	VR-14
HAdV-D15	VR-16
HAdV-D20	VR-255
HAdV-D23	VR-258
HAdV-D24	VR-259
HAdV-D25	VR-223
HAdV-D27	VR-1105
HAdV-D28	VR-226
HAdV-D29	VR-272
HAdV-D30	VR-273
HAdV-D32	VR-625
HAdV-D33	VR-626
HAdV-D38	VR-988
HAdV-D39	VR-932
HAdV-D42	VR-1304
HAdV-D44	VR-1306
HAdV-D45	VR-1307
HAdV-D47	VR-1309
HAdV-D51	VR-1603
